# Mobile Health Interventions in Gestational Diabetes Mellitus and Pregnancy Outcomes: A Scoping Review

**DOI:** 10.7759/cureus.114230

**Published:** 2026-08-09

**Authors:** Roopa R Mendagudali, Shailaja S Patil, Pratibha Rao, Ravi S Kurle

**Affiliations:** 1 Community Medicine, Gulbarga Institute of Medical Sciences, Kalaburagi, IND; 2 Community Medicine, Public Health, Shri BM Patil Medical College and Hospital, BLDE Deemed to be University, Vijaypura, IND; 3 Community Medicine, Mahadevappa Rampure Medical College, Kalaburagi, IND

**Keywords:** gdm therapy and monitoring, gestational diabetes, mhealth, mobile health, pregnancy

## Abstract

Gestational diabetes mellitus (GDM), one of the rising epidemics in pregnancies worldwide, is defined as glucose intolerance that is first recognised during pregnancy. GDM is associated with adverse maternal and fetal outcomes, including pre-eclampsia, caesarean delivery, and macrosomia. Mobile health (mHealth) interventions have emerged as promising tools for managing GDM, enabling remote monitoring, education, and communication. Hence, the present study was undertaken to map the nature of the use of mHealth interventions in GDM and report pregnancy outcomes based on the available literature.

The scoping review was conducted following a systematic search for studies in PubMed, ScienceDirect, and Google Scholar databases in the last five years (2019-2024) that investigated an mHealth app intervention for mothers with GDM and reported maternal or fetal outcomes. Both randomised controlled trials (RCTs) and observational studies were included.

A total of seven studies were included in this review, of which four were randomised trials and three were observational studies. The types of mHealth interventions varied widely, including mobile applications, SMS-based platforms, wearable devices, and teleconsultation systems. An RCT (China, 2019) reported that a nurse-guided smartphone app significantly improved glucose control and reduced weight gain in patients with GDM compared with usual care. Further, a study (Sweden, 2021) reported improved glycaemic control and compliance. Another trial (Slovenia, 2023) showed that a comprehensive telemedicine programme led to better postprandial glucose control and fewer caesarean deliveries. A Swiss trial (MySweetheart trial, 2024) combining app-based and telehealth coaching reduced gestational weight gain.

The study revealed that app-based intervention such as mHealth showed potential to support glucose monitoring, weight management, improvement in pregnancy outcomes, and patient engagement in GDM.

## Introduction and background

Gestational diabetes mellitus (GDM) is a form of glucose intolerance that is first recognised during pregnancy. It is a health condition in which women without previously diagnosed diabetes exhibit high blood glucose levels during pregnancy [1]. It affects 7-18% of pregnancies worldwide, and poor glycaemic control in gestational diabetes would increase the risk of complications and is associated with adverse maternal and foetal outcomes, including pre-eclampsia, caesarean delivery, and macrosomia [1,2]. Women with GDM are at significantly higher risk of developing type II diabetes mellitus and cardiovascular morbidity later in life [3,4].

The concept of mobile health (mHealth) was proposed to support the delivery of healthcare systems worldwide. mHealth involves the use of Information and Communication Technology to provide or deliver healthcare services. The Global Observatory for eHealth (GOe), a WHO subsidiary, devoted an entire section of its global survey to mobile health (mHealth) in 2009. mHealth was defined by GOe as medical and public health practice supported by mobile devices such as mobile phones, patient monitoring devices, personal digital assistants, and other wireless devices [5].

mHealth mainly involves the employment of the basic functionality of mobile phones in the delivery of healthcare services, and these functions include voice calls and short messaging services, also known as Short Messaging Services (SMS), as well as more complex functionalities and applications as part of mobile phones or as independent wireless devices [6].

The primary management of GDM includes dietary and physical activity modifications, as well as self-monitoring of plasma glucose, and is primarily delivered through traditional education approaches and face-to-face consultation [7,8]. These traditional interventions are resource-intensive and may be limited in scalability. Mobile health (mHealth) interventions have emerged as promising tools for managing GDM, enabling remote monitoring, education, and communication [9]. Adoption and implementation of mHealth strategies are only intended to support existing healthcare delivery systems. A systematic review of the economic evaluation of mHealth solutions, conducted mostly in upper- and upper-middle-income countries, concluded that mHealth is cost-effective [10]. mHealth-related harms range from loss of privacy, loss of reputation, poor quality of data, poor lifestyle decisions, and inappropriate but reversible clinical actions to inappropriate and irreversible clinical actions [11].

GDM is a significant public health concern, with increasing prevalence globally. The advent of mobile health (mHealth) technologies, particularly mobile applications, offers innovative solutions for managing GDM. Currently, there are limited, heterogeneous, and evolving data on mHealth interventions, especially in the management of GDM. Hence, this study aims to map the nature of the use of mHealth interventions in GDM and the reported pregnancy outcomes from the available literature. The outcome of this review will provide data to advise clinicians, diabetes nurse educators, and dieticians on the benefits and compelling need for implementing mHealth.

This work was previously presented as a conference oral presentation at COGNI Sense+ 2025, the International Conference hosted by the School of Public Health and JSS Medical College, Mysuru, on July 10-11, 2025.

This article was previously posted to the Research Square preprint server on April 9, 2026; however, the preprint is not pending full publication.

## Review

Methods

Study Design

The scoping review reporting followed an adaptation of the PRISMA-ScR (Preferred Reporting Items for Systematic Reviews and Meta-Analyses extension for Scoping Reviews) reporting guidelines [12].

Study Selection

The third author downloaded and imported references into the online platform Rayyan [13]. Titles and abstracts were first screened against the inclusion criteria by the first author, and uncertainties about article inclusion were referred to the second and fourth authors for a decision. A third review was conducted independently by the first and third authors, who screened a subset (5%) of titles and abstracts of studies for eligibility to ensure that the inclusion criteria were consistently applied.

Eligibility Criteria

Studies were included if they investigated mHealth interventions targeting women diagnosed with GDM, reported maternal or foetal clinical outcomes, and were primary (empirical) research published in English in a peer-reviewed journal. Both randomised controlled trials (RCTs) and observational studies were included. Studies such as reviews of any kind, non-empirical research (e.g., case studies), and conference abstracts were excluded. No formal quality or risk-of-bias assessment was undertaken, and no appraisal was performed, as this is a scoping review design.

Search Strategy

A systematic search was conducted in PubMed, ScienceDirect, and Google Scholar for literature published between June 1, 2019, and May 31, 2024. MeSH keywords included “gestational diabetes,” “GDM,” “mHealth,” “mobile health,” “maternal outcomes,” and “foetal outcomes.” The Boolean operators used for the search were as follows: (“gestational diabetes”[Title/Abstract] AND “mHealth”[Title/Abstract]) OR (“maternal outcomes”[Title/Abstract]) OR “foetal outcomes”[Title/Abstract] AND (“GDM”[Title/Abstract] OR “mobileHealth”[Title/Abstract] OR “Maternal Outcomes”[Title/Abstract] OR “foetal outcomes”[Title/Abstract]).

Data Extraction and Synthesis

**Figure 1:**
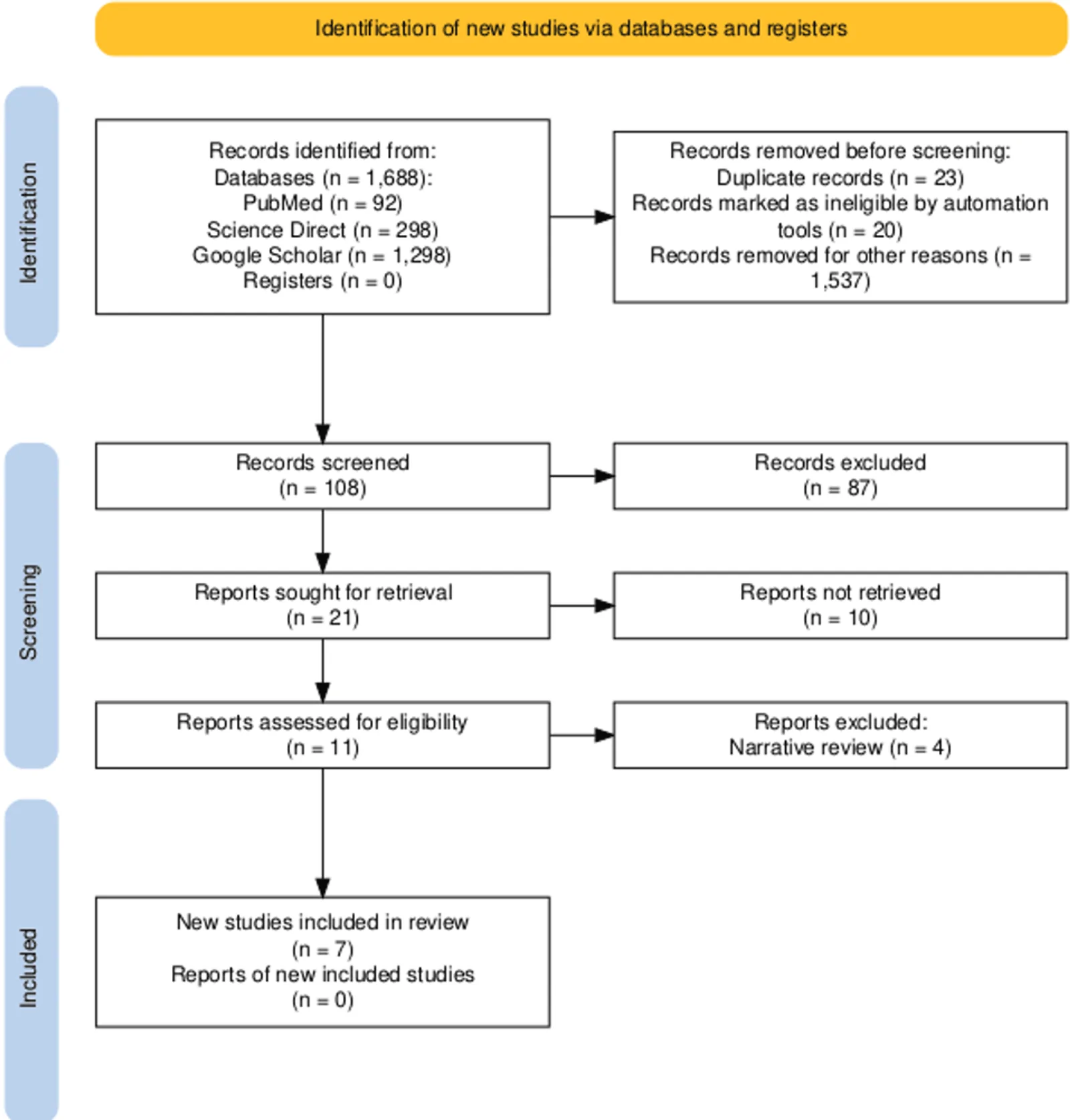
Flow diagram of selection and extraction strategy

Two reviewers independently extracted data from the studies, including authors, year of publication, study design, setting, sample size, recruitment site, stated theoretical approach, data collection method, and findings, into a custom template developed in Microsoft Excel (Microsoft Corporation, Redmond, Washington). Disagreements were resolved by discussion. We compiled a summary table of study characteristics. Results were organised by outcome domains (glucose control, weight, engagement, and maternal/foetal outcomes). No quantitative pooling or risk-of-bias assessment was undertaken. As the results were reported as a narrative summary of heterogeneous interventions, no formal meta-analysis was conducted. Figure 1 shows the flow diagram that conforms to the PRISMA 2020 standards for the review [14]. 

Results

A total of 1688 records were identified after a systematic search. The flow diagram (Figure 1) shows the study selection and screening process in accordance with PRISMA guidelines. After excluding duplicate studies and applying ineligibility criteria such as conference abstracts, review studies, studies with only protocols, screening for GDM, and access to only abstracts, 108 studies were screened by title and abstract. A total of 87 articles were excluded due to incorrect study designs, study populations, or outcomes. The remaining 21 articles were searched for full text; 10 were unavailable, and four were excluded as narrative reviews.

A total of seven studies were included in the review, of which four were randomised trials and three were observational studies. The types of mHealth interventions varied widely, including mobile applications, SMS-based platforms, wearable devices, and teleconsultation systems. As presented in Tables 1, 2, the included studies comprised randomised controlled trials, mixed-methods studies, and a cross-sectional survey conducted between 2019 and 2024 that examined mobile health (mHealth)-supported interventions for GDM.

**Table 1:** Characteristics of included studies GDM: gestational diabetes mellitus, GWG: gestational weight gain, RCT: randomised controlled trial.

Study	Design (N)	Population	Intervention	Comparator	Key Outcomes
Guo et al. [15]	RCT (N = 124)	Women with GDM	mHealth group: nurse’s online guidance via app + routine care (13 weeks)	Control: standard outpatient GDM care	Patient compliance, blood glucose (fasting/postprandial), HbA1C, GWG, maternal/neonatal outcomes
Sandborg et al. [16]	RCT (N = 305)	Pregnant women with GDM	HealthyMoms smartphone app for diet/activity counselling	Standard prenatal care	Gestational weight gain, diet, physical activity, body fat, glycemia
Munda et al. [17]	RCT (N = 106)	Women with GDM (diagnosed < 30 weeks)	App for glucose logging + monthly video consultations	Standard GDM care (monthly in- person visits)	Glycaemic control (postprandial excursions), GWG, LGA, preterm, caesarean section
Quansah et al. [18]	RCT (N = 211)	Women with GDM (24–32 weeks)	Complex lifestyle/psychosocial program: 4 prenatal visits, 4 postpartum visits, peer support, + tele-coaching via phone/app	Active usual care (per guidelines)	Maternal weight (gestational and postpartum), depression score, body fat, glycaemic indices, GWG, need for glucose- lowering meds
Bogaerts et al. [19]	Mixed- methods (N = 50)	Pregnant and interpregnancy women with overweight/obesity	INTER-ACT app + coaching sessions (lifestyle monitoring and messages)	Usability study	User interface feedback, app usefulness, and user engagement
Surendran et al. [20]	Mixed-methods follow-up (N = 170 users + 14 interviews)	Women with GDM (participants in an RCT)	Automated mHealth app- based lifestyle coaching (after RCT)	App usage study	App usage logs (meal, weight entries), user perceptions of app usefulness
Yu et al. [21]	Cross-sectional survey (N = 609)	Pregnant/postpartum women and HCPs	Survey of needs	–	Acceptance of digital GDM management, desired features

**Table 2:** Summary of Intervention Outcomes Across Studies GWG: gestational weight gain, NA: not applicable.

Study	Intervention Type	Improved Glucose Control	Reduced GWG	Improved Compliance	Lower Caesarean/Complications
Guo et al. [15]	App + Nurse Guidance	Yes	Yes	Yes	Yes
Sandborg et al. [16]	App Only	Yes	No	Yes	Not reported
Munda et al. [17]	Telemedicine (App + Video)	Yes	Yes	Yes	Yes
Quansah et al. [18]	App + Lifestyle + Psychosocial	Yes	Yes	Yes	Not reported
Bogaerts et al. [19]	App + Coaching (Pilot)	NA	NA	Yes	NA
Surendran et al. [20]	App (Usage Study)	NA	NA	Yes	NA
Yu et al. [21]	Survey (Digital Preference)	NA	NA	NA	NA

Interventions varied across studies; one used a nurse-guided mHealth app plus routine care; another used an app plus monthly video visits to replace in-person care; another combined app-based coaching, face-to-face counselling, and peer support. Control groups received standard prenatal or diabetes care in all trials. Outcomes measured included blood glucose (fasting, postprandial, HbA1c), gestational weight gain, treatment adherence, and birth outcomes (caesarean rate and neonatal weight). The pilot and surveys focused on usability, user satisfaction, and preferences.

Randomised controlled trials evaluated the effectiveness of app-based or digitally supported lifestyle and glucose-monitoring interventions. Guo et al. (2019) reported improved glycaemic outcomes among women receiving nurse-guided mHealth support in addition to routine care [15]. Sandborg et al. (2021) assessed a smartphone application that provided diet and physical activity counselling and observed improvements in lifestyle behaviours, although no significant difference in gestational weight gain was observed [16]. Munda et al. (2023) found that an intervention combining glucose-logging applications with scheduled video consultations was associated with improved glycaemic control and reduced need for pharmacological therapy [17]. Quansah et al. (2024) evaluated a multicomponent intervention incorporating tele-coaching, peer support, and extended prenatal and postpartum follow-up, reporting improvements in metabolic and psychosocial outcomes [18].

Mixed-methods studies explored feasibility, acceptability, and user experiences. Bogaerts et al. (2020) and Surendran et al. (2021) reported high user engagement, perceived usefulness, and sustained adoption of lifestyle behaviours [19,20]. A cross-sectional survey by Yu et al. (2024) identified user and provider needs for personalised, clinician-supported digital interventions. A total of 57.1% had experience using a mobile application for health management, and 58% reported unhealthy eating habits and overeating. The rate of regular exercise was only 28% [21]. 

Maternal Outcomes

Glycemic control: Guo et al. found that women using the mHealth app had significantly better glucose control than the control group. The mHealth group had fewer above-target glucose readings (fasting and 2 h postprandial) and lower pre-delivery HbA1c [15]. Munda et al. reported that the telemedicine group had a lower percentage of postprandial measurements above the glycaemic target (10.4% vs 14.6%; p = 0.015) than the standard care group [17]. The average postprandial glucose was lower in the telemedicine group (5.6 ± 0.3) than in the standard care group (5.9 ± 0.4; p = 0.004). In Quansah et al.’s study, there were no significant differences in fasting glucose or HbA1c at follow-up, suggesting that the complex intervention did not further improve glycaemia beyond usual care (Tables 2, 3) [18]. 

**Table 3:** Mean Fasting, Postprandial Glucose, and HbA1c Levels

Study	Fasting Glucose (mmol/L) (Intervention)	Fasting Glucose (mmol/L) (Contol)	Postprandial Glucose (mmol/L) (Intervention)	Postprandial Glucose (mmol/L) (Control)	HbA1c (%) (Intervention)	HbA1c (%) (Control)
Guo et al. [15]	4.9	5.4	5.6	6.1	4.7	5.3
Sandborg et al. [16]	4.8	4.8	Not reported	Not reported	Not reported	Not reported
Munda et al. [17]	5.0	5.3	5.6	5.9	5.2	5.1
Quansah et al. [18]	5.1	4.9	Not reported	Not reported	5.2	5.0

Weight gain and diet adherence: Multiple studies reported improved adherence to dietary recommendations and appropriate gestational weight gain. Guo et al. found that weight gain in the mHealth group was significantly lower (3.2 ± 0.8 vs 4.8 ± 0.7, p < 0.001) and that app-based monitoring and feedback helped reduce excessive weight gain [15]. Quansah et al. observed a statistically significant decrease in gestational weight gain since GDM diagnosis (-1.20 kg; 95% CI, -2.14 to -0.26) and in weekly weight gain throughout pregnancy (-0.14 kg; 95% CI, -0.25 to -0.03), i.e., women in the intervention group gained about 1.2 kg less than controls and had slower weekly weight gain [18]. Munda et al. did not report differences in weight gain between the telemedicine and control groups, suggesting no major effect (10.7 ± 4.5 vs 11.3 ± 5.6, p = 0.551) [17]. Sandborg et al. reported no difference in GWG [16].

Health behaviour and engagement: Most mHealth interventions demonstrated higher user engagement and satisfaction (Table 4). Guo et al. reported that the mHealth group demonstrated higher patient compliance (83.3 ± 12.5%) than the control group (70.4 ± 10.1%), with a significant difference (p < 0.001) [15]. Surendran et al. reported that only 34% of users logged at least one meal, averaging 35 meals over eight weeks, indicating low engagement with the diet-tracking component due to issues with food-item wording and database limitations. Participants reported that the weight-tracking component was easier to use, with an average of 1.85 (SD = 1.60) weight values logged per week [20] (Table 4). The application promoted self-awareness in food choices, with 43% of users mentioning that automatic coach messages motivated behaviour. Qualitative data revealed that some participants did not find the educational lessons useful because health information was readily available online.

**Table 4:** App Engagement Metrics [20]

Metric	Mean	SD
Meal logs per week	0.88	0.99
Weight logs per week	1.85	1.6

Bogaerts et al. reported that 90% of the participants indicated that an app would help them to maintain a healthy lifestyle. Among the 50 women, 46 (92%) were eager to monitor their calorie intake, and 28 (56%) were eager to monitor their physical activity goals using an app or a diary. Pregnant women preferred graphic displays, weekly notifications, and support messages tailored to their goals. Participants reported the need for mHealth as an addition to face-to-face contact, suggesting a complementary role [19]. 

Fetal Outcomes

Birth weight: Munda et al. reported no significant differences in preterm birth or large-for-gestational-age infants [17]. Guo et al. reported that mHealth reduced the rate of neonatal macrosomia (6.3% vs 10%, p = 0.295), though this difference was not significant [15].

Gestational age at delivery: Guo et al. reported that gestational age at delivery in the mHealth intervention group was higher, though not significantly (37.8 ± 2.5 vs 38.1 ± 1.6%, p = 0.654) [15]. Munda et al. also reported no difference in gestational age at delivery (39.3 vs 39.4, p = 0.581) [17].

Delivery characteristics: Munda et al. reported a significantly lower caesarean section rate in the telemedicine group (17.3% vs 35.3%, p = 0.038), whereas Guo et al. reported a lower number of caesarean sections, but this difference was not statistically significant (33.3% vs 25%, p = 0.352) [15,17]. They also reported that there were no differences between the groups in any other delivery characteristics, such as normal delivery, instrumental delivery, or episiotomy rates.

Neonatal hypoglycaemia: Guo et al. reported that mHealth reduced rates of neonatal hypoglycaemia and macrosomia, though not significantly (1.6% vs 3.3%, p = 0.185) [15].

Discussion

The present scoping review, conducted using diverse study types predominantly originating in high-income countries, found a surge in digital health research in affluent nations.

Glycaemic Control

The study indicates that mobile health-supported interventions consistently improve glycaemic control among women with GDM, thereby suggesting the potential of mHealth in maternal hyperglycaemia. Across randomised controlled trials, mHealth interventions were associated with reductions in fasting and postprandial glucose levels and, in some cases, lower HbA1c values prior to delivery, particularly when digital tools were integrated with clinician or nurse guidance [15,17]. These findings also suggest that postprandial hyperglycaemia plays a dominant role in excessive foetal glucose exposure and subsequent adverse outcomes [1,3]. Interventions including structured professional feedback in nurse-guided smartphone applications or app-based glucose logging combined with video consultations demonstrated superior glycaemic outcomes compared with app-only approaches [15,17]. This suggests that mHealth platform apps act most effectively as extensions of conventional diabetes care rather than as standalone self-management tools, supporting the complement, not the replacement, of existing healthcare delivery systems [9].

Gestational Weight Gain and Dietary Adherence

The total and weekly GWG were reduced in studies that combined dietary monitoring, behavioural coaching, and frequent feedback [2,15,18]. However, some of the trials reported no significant differences in GWG [16,17], highlighting heterogeneity in interventions, baseline maternal characteristics, and behavioural adherence. These findings support the potential of mHealth interventions to improve metabolic outcomes while enhancing the scalability and continuity of GDM care [9,15].

Health Behaviour and Engagement

Behavioural adherence is a critical mediator of mHealth interventions across the studies. Self-monitoring of glucose, weight, diet, and physical activity was consistently higher among women receiving mHealth-supported interventions than among those receiving standard care [15,20]. Improved compliance rates observed in intervention groups support the role of digital tools in facilitating behavioural regulation that requires sustained lifestyle modification throughout pregnancy. However, engagement varied substantially across the app components. Weight logging demonstrated higher and more sustained use than dietary tracking, likely reflecting usability challenges associated with detailed food entry. Qualitative findings highlighted limitations in food databases, unclear terminology, and time constraints as barriers to consistent dietary logging, despite recognition of the importance of dietary logging for glycaemic control [20].

Automated feedback and motivational messages were identified as facilitators of engagement, promoting self-awareness and reinforcing healthier choices [19,20].

Interestingly, some participants perceived educational content as superfluous, suggesting that future mHealth interventions should prioritise personalised, actionable insights in educational modules [20,21]. Women expressed a clear preference for mHealth applications as adjuncts to face-to-face care rather than as replacements, reinforcing the hybrid model of care in GDM reported by usability and preference studies [9,19].

Maternal and Foetal Outcomes

The randomised trials reported a lower caesarean section rate among women managed via mHealth interventions, suggesting that improved metabolic monitoring and timely clinical adjustments may reduce the need for obstetric interventions [17]. In contrast, effects on neonatal outcomes, including birth weight, gestational age at delivery, macrosomia, and neonatal hypoglycaemia, varied and were often statistically non-significant [15,17].

Limitations of the Study

Our review systematically organised diverse evidence from multiple countries. However, it is limited by relying on only the seven included studies. The study revealed encouraging findings, but considerable heterogeneity was observed in intervention design (different apps, coaching intensity, and telemedicine components), duration, intensity, outcome measures, and outcome reporting, which may limit cross-study comparisons. Most studies were conducted in high-income settings, despite the growing burden of GDM in resource-limited, low- and middle-income countries, where these interventions could provide scalable digital solutions. Risk of bias cannot be assessed as this is a scoping review. The observational and qualitative studies offer context but no efficacy data. We also note a lack of long-term follow-up on maternal or foetal outcomes (beyond birth) in these studies.

The potential harms of mHealth, including risks to data privacy and quality, have been acknowledged in the literature. Some participants struggled to use mHealth tools effectively due to a lack of digital literacy, suggesting that adequate training and/or a tour guide should be included during app development.

Further, few interventions reported theoretical or behavioural frameworks in their study design, limiting practical understanding and reproducibility. With appropriate design and implementation, these technologies can improve clinical outcomes for both mothers and their infants.

## Conclusions

The study findings show that mHealth interventions in GDM have a positive impact on glycaemic control, gestational weight gain, and pregnancy outcomes. The apps that allow self-monitoring of glucose and deliver automated coaching have shown promise in improving control and engagement. However, no single app has emerged as best-in-class. The mixed results suggest that mHealth is an adjunct, not a replacement, for comprehensive GDM care.

Future work should prioritise theory-driven intervention development, standardised outcome measures, user-centred design, equitable access, ease of use, and practical trials embedded within existing routine antenatal care. The integration of mHealth tools into existing healthcare systems will be critical to maximise effectiveness and sustainability.
